# Editorial: Investigating the role of microorganisms in ecosystems and their interactions with the humans, animals, plants, and environment interface

**DOI:** 10.3389/fmicb.2025.1710906

**Published:** 2025-11-03

**Authors:** Wen-Jun Li, Juan M. Gonzalez, Bhagwan Narayan Rekadwad

**Affiliations:** ^1^State Key Laboratory of Biocontrol, Guangdong Provincial Key Laboratory of Plant Stress Biology, School of Life Sciences, Sun Yat-sen University, Guangzhou, China; ^2^State Key Laboratory of Ecological Safety and Sustainable Development in Arid Lands, Xinjiang Institute of Ecology and Geography, Chinese Academy of Sciences, Urumqi, China; ^3^Institute of Natural Resources and Agrobiology, Spanish National Council for Research, IRNAS-CSIC, Sevilla, Spain; ^4^MicrobeAI Lab, Division of Microbiology and Biotechnology, Yenepoya Research Centre, Yenepoya (Deemed to be University), Mangalore, Karnataka, India

**Keywords:** endophyte communities, bio-priming, gut bacterial community, root-zone microbial communities, phycosphere of marine macroalgae, vaginal microbiota, antibiotic-corticosteroid treatment, phylogenetic diversity

Microbial species that live with, within, and interact with us have been of upmost importance to us for three primary reasons. The microorganisms are ubiquitous and play significant roles in shaping Earth's biogeochemical cycles, cellular and organellar wellbeing, and have noticeable applications in health. Their diminutive size often keeps them unnoticed in beneficial cycles or in a mutualism, unless noticeable health hazards are reported or catastrophic events occur. The multifaceted interaction between microorganisms and other living things, including humans, animals, and plants, at various internal and external environment interfaces, indicates their contribution to maintaining health, environmental balance, and assistance in supplying or directly producing various biomolecules and products. Moreover, published reports confirm that microbial communities are involved in combat with pathogenic microorganisms, developing control strategies, and the discovery of natural products.

On the other hand, various microorganisms (pathogenic bacteria, viruses, and protozoans) lead to serious disease conditions in humans and animals under different scenarios. Additionally, they may act as causative agents in initiating the development of neurodegenerative diseases. The latest research findings indicate that microorganisms are involved in climate and spatiotemporal changes and can be exploited for their applications in all fields of biotechnology.

This Research Topic presents a collection of articles that cover various sections related to current approaches employed in biotechnology, such as green (agricultural), red (all aspects of medical and health, including female reproductive health), blue (marine), gray (environmental), and gold (bioinformatics).

The Research Topic presents interesting roles of microorganisms and their interaction with living things at the environmental interface. To begin with, Zhu et al. presented an article dealing with root-zone microbial communities associated with *Artemisia ordosica* Krasch. They have carefully described different successional stages in Mu US Sandy Land using a combined metagenomics and culturomics approach. High-throughput analysis led to the disclosure of Proteobacteria (46.43%) along with a considerable population belonging to the rarely cultivated Deinococcota (a phylum of radioresistant and thermophilic taxa). This study offers pathways for targeted use and brings practical solutions for bioremediation.

Moreover, spatiotemporal diversity compositions from Hainan Island investigated by Li et al. have a remarkable presence of distinct endophytic fungal communities (*Lophiotrema, Lophiostoma, Neournula, Pseudallescheria, Graphiola, Symmetrospora, Phaeosphaeriopsis, Mycosphaerella, Cladosporium, Fusarium*, and *Trichoderma*), facilitating the transport of flavonoids toward and reducing autotoxic effects, aiding in higher production of resin in *D. cambodianaa* and enhancing the defense system. Some other species, such as *Cordyceps cicadae* associated with two insects, *Macrosemia pieli* and *Platypleura kaempferi* (Chen and He), and *Alcanivorax* spp., associated with predominant soil collembolan species *Entomobrya proxima* Folsom (Yang et al.), act as potential biomarkers for understanding climate change and nurturing the production of biofertilizers, respectively. A collection of novel microorganisms with a range of applications in environmental processes and biotechnology has been represented. These microorganisms have been reported from geographically diverse areas of PR China in South Asia, including *Eudoraea algarum* sp. nov., *Maribacter algarum* sp. nov., *Brumimicrobium ulvae* sp. nov., *Ulvibacter algarum* sp. nov., *Aurantiphycus algarum* sp. nov., *Tamlana algarum* sp. nov. (Lu et al.), *Cortinarius griseoaurantinus* (subgenus Leprocybe), *Cortinarius yonganensis* (subgenus Dermocybe) (Dang et al.), *Bifusisporella magnum* sp. nov., and *Letendraea bambusae* sp. nov. (Guan, Zhao, keyhani et al.), consortium of Fusarium oxysporum (Rangasamy and Saleh) and three entomopathogenic species, such as *Albacillium fuzhouense* sp. nov., *Conoideocrella gongyashanensis* sp. nov., and *Neoaraneomyces wuyishanensis* sp. nov. (Lin et al.). These newly discovered taxa may have potential applications for pest control, sustainable agriculture practices, and the discovery of novel natural products. Other species found in the environment and human samples, which were identified through high-throughput analysis of the gut microbiome and nosocomial body parts involved in the gut-organellar axis, also exhibit antimicrobial activities against other pathogens. On the other hand, a specific combination of traditional Chinese medicine, new bitongling, plays a role in modulating gut microbiome composition. The reported microbial taxa, such as f_Mycoplasmataceae, g_Prevotellaceae_Ga6A1_group, and three other taxa, are correlated with expressed microRNAs (miR-20a-5p and miR-223-3p) that may be associated with new bitongling. Hence, new bitongling might be a potential biomarker for a holistic understanding of how NBTL influences rheumatoid arthritis pathogenesis via the gut-joint axis (Guan, Zhao, Lu et al.). Investigations by Fang et al. have shown that the Lhasa (China) skin microbiome study indicates that fungal strains such as *Malassezia, Aspergillus, Aureobasidium*, and *Penicillium* were the dominant genera and mainly associated with the population that developed melasma.

The phylogenetic diversity and non-aureus staphylococci (NASM) and mammaliicocci from the teat apices of 114 organic dairy cows were studied by Peña-Mosca et al. Genome-guided analysis for the determination of virulence genes, intramammary infections (IMI), antimicrobial peptides (AMPs), and resistance genes indicates that the investigated cows have harbored multiple NASM, and each has at least one AMP gene. All detected strains of *Staphylococcus aureus* have higher virulence and lower species-specific prevalence than other strains. Nowadays, antibiotics, used either in combination with or without steroids, are widely used in veterinary care and the treatment of mammals other than cows, goats, and sheep, such as horses. de Bustamante et al. carried out a longitudinal and blinded study to examine the effect of medication antibiotics (with or without corticosteroids). The composition of the microbiome was dominated by Proteobacteria at baseline (0 days), and after 30 days, it returned to near-baseline levels in all horses. It was also observed that the microbiota had returned to its original state after 3 weeks, which is near the baseline. This confirms that the microbiome can restore its original composition, which is directly proportional to the presence of antibiotics.

Recent advances in microbiome research have successfully investigated and presented a structured analysis of microbial communities, as well as enhanced understanding of various ecological processes and the connection between microbiomes and various internal and external organs. However, the outer environment and internal environment of organs play a pivotal role in the existence of various unexplored microbial species, as well as those with differential potentials (in the case of already known) microbiota. Nowadays, recent discoveries in reproductive health have been explored for the impacts and roles of microbiomes. Zhai et al. investigated the dynamics of microbial communities inhabiting the vagina at different hormonal states during all reproductive phases in 150 subjects, divided into five groups. This allowed for an understanding of microbial community composition of the follicular phase (group 1), luteal phase (group 2), early pregnancy (group 3), lactation phase (group 4), and menopause (group 5). Each group consists of 30 subjects. It was recorded that the first two phases (groups 1 and 2) were dominated by Firmicutes, followed by a noticeable increase in Bacteroidetes, Actinobacteria, and Proteobacteria during lactation and menopause. Surprisingly, the phylum Crenarchaeota was found in abundance in the last two phases. From the beginning to the end, *Lactobacillus* was dominant among all genera throughout all phases, followed by an increased dominance of *Prevotella*, then *Streptococcus, Ralstonia, Gardnerella, Dialister*, and *Pseudomonas* in the later phases. Zhang et al., in another independent study, have proved that there could be a vaginal microbiome composition that may be a cause of preterm birth in babies of Chinese women. Zhang et al. has clearly outlined that the relative abundance of harmful bacteria, such as *Gardnerella, Ralstonia, Atopobium*, and *Sneathia* was significantly increased during the pregnancy. This study has potential as a prospective predictor for preterm birth of babies, which may be caused by the increased dominance of harmful microbiota. Indeed, the outcome of this study acts as a theoretical foundation for planning treatment strategies to avoid preterm births.

Globally, microorganisms play major roles in human, animal, and plant wellbeing and development. Despite their microscopic size, they constitute a pillar that supports the functioning of environments and all their macroscopic living components.

